# Editorial: Bacterial population heterogeneity, stress response and antibiotic tolerance

**DOI:** 10.3389/fcimb.2025.1754314

**Published:** 2025-12-11

**Authors:** Abhishek Subramanian, Krishna Kurthkoti, Vittoria Mattioni Marchetti, Srinivasan Vijay

**Affiliations:** 1 Department of Biotechnology, Indian Institute of Technology Hyderabad, Telangana, India; 2 Chanakya University, School of Biosciences, Bengaluru, India; 3 Unit of Microbiology and Clinical Microbiology, Department of Clinical-Surgical, Diagnostic and Pediatric Sciences, University of Pavia, Pavia, Italy; 4 Department of Microbial Pathogenesis and Immunology, Texas A&M University Naresh K. Vashisht College of Medicine, College Station, TX, United States

**Keywords:** antibiotic resistance, antibiotic tolerance, pharmacokinetics, pharmacodynamics, heteroresistance, bio-films, carbapenem resistance

The emergence of antibiotic resistance is a critical challenge for infectious disease control and global health, resulting in treatment failure and increased mortality (Abbas et al., 2024). Multiple independent and synergistic mechanisms enable bacteria to survive antibiotic treatment, relapse, and evolve resistance (Sulaiman and Lam, 2022; Datta et al., 2024). Intrinsic factors—such as population heterogeneity that generates persistent or tolerant subpopulations, and stress responses that promote genetic variation—play a central role in the development and stepwise progression of antibiotic tolerance towards resistance (Brauner et al., 2016; Levin-Reisman et al., 2017; Balaban et al., 2019). While traditional diagnostic and therapeutic approaches focus on resistance detection (WHO, 2025), there is growing emphasis on understanding molecular mechanisms underlying bacterial stress response and tolerance (Nandakumar et al., 2014; Vijay et al., 2024), and employing novel antibiotic combinations and potentiators to improve outcomes (Ilchenko et al., 2024).

In the Research Topic, a comprehensive review by Alikhani et al. highlights the importance of pharmacokinetic (PK) and pharmacodynamic (PD) principles in antibiotic therapy. The authors detail how the susceptibility of pathogens to antibiotics is defined alongside PK processes—absorption, distribution, metabolism, and elimination—which guide optimal dosing, minimize side effects, and improve patient outcomes. PD considerations address the mode of action of drugs and offer a framework for classifying antibiotics by their PK/PD relationships. Drug dosing strategies are refined to either maximize peak concentration relative to minimum inhibitory concentration (Cmax/MIC) or duration based sustain therapeutic drug levels (area under the curve) above MIC (AUC (area under the curve)/MIC), depending on the mechanism of action. The review underscores the challenges in antibiotic development and exploring new strategies for effective treatment against resistant organisms.

Recent research has illuminated how phenotypic plasticity, triggered by transient physiological states and stress, contributes to antimicrobial tolerance and heteroresistance. Zheng et al. reveal that species-specific thermal environments modulate fluconazole tolerance in fungal pathogens. Whereas, Luo et al. demonstrate that non-genetic, transcriptionally driven mechanisms lead to reversible heteroresistance to imipenem in *Escherichia coli*. Such findings mark a shift in understanding microbial resilience, opening up possibilities for targeted interventions.

Heteroresistance describes a resistance phenotype where subpopulations of bacteria within a clonal isolate show different levels of susceptibility to an antibiotic (Band and Weiss, 2019). Luo et al. show that a clinical *E. coli* strain can develop imipenem heteroresistance via gene regulation without prior antibiotic exposure. Using population analysis profiles (PAPs), they document the reversion of heteroresistant subpopulations to susceptibility after removal of drug pressure, along with rapid re-emergence upon low-level drug exposure. The instability and reversibility of this phenotype have dynamic gene regulation as a key mechanism. Additionally, heteroresistance correlates with the cohesion of the outer cell membrane and biofilm formation, which can further impact antibiotic resistance. These observations advocate for clinical settings to eliminate both residual antibiotics along with residual bacteria and suggest that secondary drugs may be effective against non-genetic heteroresistant populations.

Zheng et al. explore temperature-dependent phenotypic fluconazole tolerance in fungal species, showing that environmental temperature can alter drug susceptibility. For instance, *C. albicans* retains fluconazole tolerance at 37°C whereas *S. cerevisiae* maintains tolerance at 30°C but develops resistance at 37°C through the formation of petite mutants lacking respiratory competence and unable to grow on non-fermentable carbon sources. Their results also show that petites upregulate efflux genes, which contributes to fluconazole resistance, and downregulate genes involved in ergosterol biosynthesis. In addition, stress-response components such as calcineurin and Hsp90 contribute to fluconazole tolerance. These insights into species-specific drug responses can contribute to effective antifungal treatment strategies.

Biofilms represent a special ecological niche with genetic and metabolic heterogeneity and augmented tolerance and resistance to environmental stresses, including antibiotics (Vareschi et al., 2025). The work of Salini et al. investigates genetic diversity within biofilms of non-pathogenic *Mycobacterium smegmatis*, a model relevant to *M. tuberculosis*, the causative agent of tuberculosis. Although the concept of mycobacterial biofilms is longstanding, their clinical relevance for disease progression and drug resistance has only recently gained recognition. Salini et al. show that during biofilm maturation, metabolic changes—high NADH and reduced ATP—promote reactive oxygen species (ROS) accumulation. Transcriptome analysis confirms that ROS detoxification enzymes are downregulated, sustaining high ROS levels, which trigger DNA damage and the SOS response. This activates the mutasome (composed of DnaE2, ImuA’, and ImuB), elevating mutagenesis; loss of *dnaE2* diminishes both mutagenesis and competitive fitness. Their findings indicate that biofilms not only shelter bacteria from stress but also act as sources of genetic diversity, underpinning the frequent association of drug tolerance and resistance in tuberculosis.

Bringing these mechanisms into clinical perspective, Li et al. analyse the interplay between fungal infections and carbapenem-resistant gram-negative bacilli (CRGNB) infection outcomes. In a large retrospective cohort (n=2,273), they demonstrate bidirectional risk—fungal infections raise susceptibility to CRGNB and vice versa. They identify overlapping risk factors such as male gender, ICU admission, invasive surgery, cancer, ventilator support, drainage tubes/catheters, and prior cephalosporin/carbapenem use with susceptibility to both infections. Notably, fungal co-infections are linked to increased mortality in patients with CRGNB, especially those suffering from carbapenem resistant *Klebsiella pneumoniae* and *Acinetobacter baumannii*. Furthermore, fungal infections often precede CRGNB emergence. This study highlights the importance of assessing co-infections in the management of antibiotic-resistant cases and the urgent need for multi-faceted clinical approaches.

Further, Li et al. review the emerging pathogen *Corynebacterium striatum*, which is associated with a wide range of severe infections—including pneumonia, bacteraemia, meningitis, joint and abdominal infections, and endocarditis. Outbreaks and respiratory manifestations have increased since the COVID-19 pandemic in both hospital and community settings. Diagnostic challenges persist, as biochemical methods are prone to misidentification, whereas molecular and mass spectrometric techniques (e.g., 16S rRNA and MALDI-TOF) improve accuracy. The virulence of *C. striatum* is driven by biofilm formation and iron uptake. Genomic studies reveal resistance mechanisms such as efflux pumps (*tetA/B*), target modifications from *gyrA* mutations, and antibiotic inactivation genes such as the aminoglycoside N – acetyl/O-phospho transferase genes (*aac(6’), aph(6’), aph(3’)*), with transposon Tn5432 conferring macrolide-lincosamide-streptogramin B resistance via *erm(X)* gene. Despite various treatments, the rise of multidrug resistant (MDR) *C. striatum* complicates optimal therapy. Current recommendations favour vancomycin monotherapy or its combination with agents like piperacillin-tazobactam for lung infections, with dalbavancin proposed for treating soft tissue cases. Niclosamide, which reduces biofilm and cell viability, is a promising alternative.

In closing, recent advances in antibiotic and antifungal resistance research reveal the intertwined effects of PK/PD principles, microbial stress responses, phenotypic adaptation, and clinical risk factors in shaping drug efficacy. Notably, non-genetic tolerance, biofilm dynamics, and environmental influences drive rapid shifts in susceptibility and the emergence of antibiotic tolerance and/or resistance. These insights foster innovation in diagnostics and therapeutic design. A comprehensive understanding of microbial resilience promises better strategies to prevent treatment failure and combat antimicrobial resistance globally.
